# New York Letter

**Published:** 1897-09

**Authors:** Ogden C. Ludlow

**Affiliations:** 2309 Seventh Avenue; New York


### CORRESPONDENCE.

### NEW YORK LETTER.

NEW YORK, August 10, 1897.

In these midsummer days the medical mind is prone to dwell upon the subject of heat prostration. Just as it has been the custom here for some years past to refer all sorts of ills to the time of "our blizzard," so it is likely to become a popular habit to ascribe many disorders to the record-breaking heated term of last August. Those who spent that portion of the summer of 1896 in the city are not likely to forget it, yet it is well for all to refresh their minds with some of the important lessons drawn from that experience. The subject has been very carefully studied by Dr. Alexander Lambert, in a paper based on over six hundred cases of sunstroke; and he shows quite conclusively that, aside from the peculiar meteorological conditions present, excesses of all kinds, and particularly indulgence in alcoholic beverages, are powerful predisposing causes of sunstroke. Regarding the weather conditions existing at that time, he quotes from the records of the observatory in Central Park. According to these, the mean humidity was 73.18 per cent., and the temperature ranged from 72 to 98 degrees Fahrenheit, being for five consecutive periods of twenty-four hours between 80 and 98 degrees Fahrenheit. The average temperature was 86.7 degrees Fahrenheit, or 10.6 degrees above the monthly mean temperature. But even these figures do not give an adequate conception of the sufferings of those who were in the city streets—not in the park. It should be remembered that during the working hours of the day the average temperature in the sun was 119.3 degrees Fahrenheit, that there was scarcely a perceptible breeze, and that the temperature remained in the vicinity of 90 degrees Fahrenheit up to midnight.

Direct exposure to this scorching sun was not necessary to produce heat prostration, although the majority of the patients had been more or less exposed in this way. The cases divided themselves clinically into three classes, viz.: (1) Heat prostration; (2) the asphyxial or milder form; and (3) the hyper-pyrexial or severe form. The prodromal symptoms were usually headache, dizziness and pain in the back. In only a few of the cases was there a cessation of perspiration. In the third class the prodromal symptoms were of longer duration, the individual sometimes being sleepless, weak and irritable for two or three days. The experience of last summer brought out very prominently one fact, viz.: that the higher body temperature alone was not the chief source of danger, for patients were admitted to hospitals with little or no elevation of temperature, or even with a sub-normal temperature, though often unconscious. Moreover, death occurred in some cases in which the temperature had ranged between 103 and 105 degrees Fahrenheit for a few hours, while, on the other hand, one individual survived a temperature of 115 degrees Fahrenheit! These facts, taken in connection with some pathological studies made by Dr. Ira Van Gieson, have led the latter to express the opinion that sunstroke is a species of auto-intoxication. Another point of interest is that the pulse does not maintain the usual relation to the body temperature, its frequency seeming to depend upon the degree of nervous shock. In most of the fatal cases, the respiratory and cardiac centers seemed to fail simultaneously. In considering the treatment of sunstroke, Dr. Lambert pointed out that drugs did not act in their usual doses—indeed, they had but little effect, until the body temperature had been reduced to 103 or 104 degrees Fahrenheit. It was found necessary in most of the cases to employ active measures for reduction of temperature three times within the first twenty-four hours. Although many of the patients became conscious within a short time after the first bath, one person, who ultimately recovered, did not regain consciousness for ten days. In the treatment of the six hundred cases considered in this paper various methods were employed, but the best results were obtained from the use of the needle spray, with water at a temperature of about 75 degrees Fahrenheit, or the ice bath 40

degrees Fahrenheit, given until the temperature fell to 103 or 104 degrees Fahrenheit, followed by a hot pack. One precaution should be taken in the use of the ice bath, and that is, that the patient be not allowed to come in contact with the ice in the bath, for in several instances a neglect of this precaution resulted in sudden cessation of the respiration and death. While in the bath all parts of the body should be constantly and vigorously rubbed, and the temperature taken at intervals of two or three minutes.

Another subject of special importance in the heated term, though ever demanding the serious and thoughtful attention of the physician, is that of infant feeding. We hear much less at the present day about the peculiar virtues of the different proprietary infant foods, and more about the "modification" of milk. Dr. Thomas Rotch, of Boston, the great apostle of "modified milk," has lost none of his earnestness in putting forward the claims of this most scientific method of adapting cow's milk to the needs of the individual infant, yet experience has led him to speak less dogmatically regarding the actual percentages required, and to lay more stress on the general principles which should govern the physician in prescribing milk in "percentages." It is of the greatest importance that the physician essaying to use modified milk should thoroughly understand these principles. This is well exemplified in a case which Dr. J. E. Winters reported, in which a failure on the part of a physician to grasp these facts led to the use of modified milk in such a form that the child taking it developed a well marked scurvy. That the scurvy was directly due to an improper modification of the milk, and not to other causes, was demonstrated by the prompt recovery of the child, without the use of any medicines, simply by properly adjusting the percentages of the constituents of the milk. This was one of many interesting points brought out in the discussion of a paper read by Dr. Rotch before the New York Academy of Medicine. Dr. Rotch said that as a result of increasing experience there was at the present time a distinct tendency among physicians to prescribe lower percentages than formerly. In his opinion, many premature infants had died because their gastro-intestinal tract had not been sufficiently developed to allow of the proper digestion of even the mother's breast milk. In most cases, such an infant would thrive better on a milk containing 1 per cent. of fat, 3 or 4 per cent. of sugar, and only 35 per cent. of proteid. Weaning should be effected gradually, the composition of milk being made to approximate more and more closely that of unmodified cow's milk—*i. e.*, fat 4, sugar 4.5 and proteid 4 per cent. In the discussion the most important made, and insisted upon especially by Dr. J. P. Crozer Griffith, of Philadelphia, and Dr. L. E. Holt of New York, was that the percentages of fat and proteid should be kept low. Some infants will not do well unless the proteid is reduced below one per cent.

Lest some doubting Thomases should still be disinclined to admit the great practical value of pasteurizing milk, as well as modifying it, Dr. S. E. Getty has just collected the statistics of the city of Yonkers, regarding the effect on the infant mortality of dispensing pasteurized milk. In a place of that size, it is much more easy to obtain the necessary data than in a larger city. His figures certainly speak well for the method, and serve to corroborate the statistics collected by Dr. Freeman in conjunction with the work of the Strauss milk depots in this city. Dr. Freeman, who is qualified to speak with authority on the subject of the pasteurization of milk, now advises that the milk be subjected to a temperature of 68 degrees C. for thirty minutes, which is sufficient to destroy the typhoid and tubercle bacilli without altering the taste of the milk.

During the past few months the Academy of Medicine has been favored with two interesting contributions to the treatment of fractures. One of them, by Dr. J. P. Fiske, discussed the ambulatory treatment of fractures of the leg—a method which was introduced into the Roosevelt Hospital in 1888 by Dr. John M. Woodbury, and which has been used quite extensively there and in the Bellevue Hospital. After a thorough reduction of the deformity, an ordinary roller bandage is applied from the toes to the tuberosity of the tibia, and the plaster of Paris is applied over this without padding. To secure the requisite elasticity and lightness, it is advised that a gauze bandage, impregnated with plaster of Paris be first applied, then a starch bandage similarly prepared, and over these the usual plaster of Paris bandages. The limb should be kept in the horizontal position for twenty-four hours, after which the patient may walk about if the splint is comfortable and there is no tendency to swelling. It has usually been necessary to wear the splint for four or five weeks, and on its removal the limb has been showered with hot and cold water several times a day. This treatment received favorable mention also from Dr. Willy Meyer, Dr. George Woolsey, Dr. H. Lilienthal and Dr. A. B. Johnson. Dr. Lilienthal said that the patient would be materially assisted in walking, and the splint prevented from breaking down, by adding to the splint a "heel" made out of a roller bandage of unbleached muslin soaked in plaster of Paris. In very oblique fractures there is some risk connected with this ambulatory treatment, particularly if employed from the beginning.

The other paper referred to was by Dr. George Woolsey, and was upon massage in the treatment of fractures. The author stated that the best results from this treatment were obtained in fractures at the ends of the bones—in other words, in just that class in which immobilization yields the worst results. He believed that a combination of the ambulant and the massage treatment constituted the very best method of managing peri-articular fractures of the leg. Bony union is obtained in about three weeks, and then the patient is allowed to get up. He is usually able to walk well in a very few days. In order to practice the massage, the limb should be placed on a sand pillow, the hand lubricated with olive oil, and the massage given methodically in the direction of the blood current. The pressure should be very slight at first, and should be gradually increased in proportion to the age of the fracture, but it should not be made at all over the seat of fracture. The massage should be given for from fifteen to thirty minutes daily, after which the limb should be placed in suitable splints.

In a recent letter I called attention to the fact that Dr. A. M. Phelps had succeeded in securing a good result in a very obstinate case of ununited fracture by a method of bone-grafting. In that case, *very fine spiculæ* of bone were inserted under the periosteum, and firm union of the fracture resulted, after all the usual methods of treatment had failed. In this connection it may be of interest to refer to a case illustrative of the unsatisfactory result that may be obtained when bone *chips* are implanted, even though the immediate result be very gratifying. The case was that of a young man who, in 1891, had had a cavity in his tibia filled up with the bone chips. The case apparently did well for five years, and then returned to St. Luke's Hospital with another abscess in this region. On cutting down upon the part, a considerable number of the bone chips were exposed and removed. They had undergone no apparent change as a result of their long stay in the tissues.

OGDEN C. LUDLOW, M.D.

*2309 Seventh Avenue.*

